# Pharmacovigilance and Adverse Drug Reactions Reporting: Healthcare Providers' Experiences from Southern Highland Tanzania

**DOI:** 10.1155/2023/5537592

**Published:** 2023-10-16

**Authors:** Dorkasi L. Mwakawanga, Manase Kilonzi, Erick G. Philipo, Aron Martine, Tusaligwe Mbilinyi, Nancy F. Kileo, Bryceson Mkinga, Cleopatra Justine Shonyella, Juma A. Mohamedi, Aurelia Clement, Davance Mwasomola, Stella E. Mushy, Nathanael Sirili

**Affiliations:** ^1^School of Nursing, The Muhimbili University of Health and Allied Sciences, P.O. Box 65001, Dar es Salaam, Tanzania; ^2^School of Pharmacy, The Muhimbili University of Health and Allied Sciences, P.O. Box 65013, Dar es Salaam, Tanzania; ^3^School of Diagnostic Medicine, Muhimbili University of Health and Allied Sciences, P.O. Box 65001, Dar es Salaam, Tanzania; ^4^Department of Pharmacy, Mbeya Zonal Referral Hospital, P.O. Box 419, Mbeya, Tanzania; ^5^School of Public Health and Allied Sciences, The Muhimbili University of Health and Allied Sciences, P.O. Box 65001, Dar es Salaam, Tanzania

## Abstract

**Purpose:**

This exploratory qualitative study aimed to analyze the experiences of healthcare providers (HCPs) in pharmacovigilance (PV) and ADR reporting in the southern highland zone of Tanzania.

**Methods:**

In 2022, an exploratory qualitative case study using in-depth interviews (IDIs) was conducted to explore the experiences of PV and ADR reporting among HCPs (doctors, nurses, and pharmacists). The study was carried out in a zonal referral hospital and a regional referral hospital of the Tanzanian southern highlands zone. Inductive-deductive thematic analysis was adopted for data analysis.

**Results:**

Participants demonstrated adequate knowledge of PV and its related activities including ADR reporting. Knowing the interactions and wrong medication dosage as sources of ADR, signs, and symptoms, stopping the drug, and treating the symptoms following ADR emerged as subthemes linked with adequate knowledge in identifying and managing ADR. Participants perceived reporting ADR as laborious, posing a subjective burden and that not all ADRs needed to be reported. The latter contributed to limited participation in ADR reporting despite that participants were conversant with both physical and online ADR reporting platforms.

**Conclusion:**

Although HCPs are well informed about PV and ADR reporting including the benefits to public health, their involvement in ADR reporting is low. In addition to the ongoing on-the-job training and regular supportive supervision for HCPs to improve the ADR practice, there is still a need to explore other strategies to be used as motives for HCPs to report ADR regularly.

## 1. Introduction

Adverse drug reaction (ADR) is a response to a drug that is harmful and unintended that occurs at doses normally used in humans for the prophylaxis, diagnosis, or treatment of disease or for the modification of physiological functions [1]. ADR is a major public health issue that significantly impacts clinical practices globally. ADR has several negative effects, such as drug-related hospital admissions, more extended hospital stays, emergency department visits, and a higher risk of mortality [2, 3]. When new medications are prescribed for symptoms resulting from another medication, which is frequently overlooked, ADR may trigger prescription cascades [4]. The main costs associated with ADRs at hospitals are wages, consumables, and medications [5]. Moreover, ADRs have several indirect costs for patients and their families such as lost workdays and/or morbidity like anxiety [6].

Pharmacovigilance (PV) refers to the science and activities aiming at reducing or preventing ADRs [7]. Under PV, there are two methods of ADR reporting, namely, active and passive surveillance [8]. Despite being a widely or most commonly used method, spontaneous reporting (passive surveillance) depends on the initiative and willingness of the reporters who are typically healthcare providers (HCPs) [9]. The dedication of HCPs to ADR reporting is the cornerstone of an effective PV framework. In Tanzania, two approaches can be used by HCPs to report ADRs to the national regulatory authority. First is through the use of a paper-based system, which uses yellow forms which are specific for reporting ADRs. Second is an electronic reporting platform, whereby HCPs report ADRs through Tanzanian Medicines and Medical Devices Authority (TMDA) website (https://www.tmda.go.tz) or as an unstructured supplementary service data (USSD)/text message by dialing *∗*152*∗*00# and by dialing the toll-free number 080011 0084 [10].

ADR reporting aims to reduce patient mortality and severe outcomes, readmissions to hospitals, overall hospital expenses, and future ADR incidences and improve the quality of patient care [11]. On the other hand, ADR monitoring and reporting programs provide information on the efficacy and safety of pharmaceutical products, initiate risk-management plans, avert the expected adverse effects, and aid in calculating the incidences of ADR [12]. In addition, it raises awareness of ADR and educates the healthcare team and patients about ADR. Medical doctors, nurses, and pharmacists are the HCPs in charge of identifying, documenting, reporting, and preventing ADR in routine clinical practice [13]. According to the literature, some ADRs can be prevented if HCPs align their prescribing with current medical guidelines and most ADRs can be avoided if HCPs explore and reconcile probable ADR contributing factors [14]. ADR reporting in Tanzania has been improved through the implementation of several strategies by the Ministry of Health through TMDA, including the introduction of PV regulations in 2018 and guidelines in 2020 and the appointment of a PV focal person in each country zone to ensure regular training of HCPs and patients' sensitization.

Despite the improved ADR reporting systems, underreporting remains the major drawback in identifying and preventing ADR in Tanzania. Several reasons for under-reporting of ADRs by HCPs have been reported including complacency, fear of legal action, guilt for causing harm to a patient, lack of awareness, insecurity about reporting suspicions of an ADR, and indifference [15]. Often, HCPs feel inadequately trained in ADR identification, reporting, and prevention. This is because they sometimes struggle to differentiate between the terms used to describe adverse drug events including adverse drug reactions, medication errors, and side effects which significantly impact ADR reporting rates [16]. We aimed to explore the knowledge and perceptions of the HCPs in PV and ADRs reporting in the southern highland zone of Tanzania.

## 2. Methods

### 2.1. Study Design and Setting

This research is part of a larger study that aimed to assess the reporting of adverse drug reactions by healthcare providers and consumers in Tanzania's southern highlands in 2022. We employed an exploratory qualitative case study using IDIs to analyze the experiences of HCPs on ADR reporting. The study was conducted at Mbeya zonal referral hospital (MZRH), a tertiary-level hospital, and Mbeya regional referral hospital (MRRH), a secondary-level hospital in the Southern highlands of Tanzania. Tertiary and secondary-level hospitals were selected assuming that the HCPs in these hospitals had the potential for knowledge acquisition regarding ADRs and prior experience with PV and ADRs reporting. In addition, the designated PV focal person works at the zonal referral hospital, where one of his/her responsibilities is to facilitate the execution of PV-related activities, including reporting ADRs at primary, secondary, and tertiary-level hospitals within the respective zone. Therefore, the two facilities were purposely selected to represent secondary- and tertiary-level hospitals that could provide a better picture of ADR reporting in Tanzania.

It is worth noting that the healthcare system of Tanzania is categorized into three levels, i.e., the primary healthcare level which includes district hospitals, health centers, and dispensary, the secondary level which includes regional hospitals which are named as regional referral hospitals (RRHs), and the tertiary level comprising the national hospital, specialized hospital, and zonal referral hospitals (ZRHs).

### 2.2. Adverse Drug Reaction-Reporting Channel in Tanzania

Adverse drug reaction reporting in Tanzania can be reported through two channels. First, from the consumer/patient directly to the regulatory authority (Tanzania Medicines and Medical Devices Authority (TMDA)) or from patient/consumer to HCPs and then the HCP report to the regulatory authority (TMDA). Second, HCPs can initiate ADR reporting to the regulatory authority (TMDA). Then, the TMDA compiles the reports and sends to the Uppsala Monitoring Center.

### 2.3. Study Participants and Recruitment

We purposefully recruited HCPs (PV focal person inclusive) based on their years of experience to participate in the study. The researchers requested the head of the pharmacy unit in each hospital to provide a list of medical doctors, pharmacists, and nurses involved in prescribing, dispensing, and administering medications and are in frequent contact with patients. The researchers identified participants with more than two years of work experience to get rich information on ADR reporting. The selected participants were also the respective hospital therapeutic committee's members believed to provide in-depth information regarding the PV and ADR reporting practices in the respective healthcare facilities. Therefore, a total sample of seven HCPs (including PV zonal representative) was invited face to face to participate in the IDIs.

### 2.4. Data Collection Guide and Procedures

We developed three semistructured interview guides for the three different healthcare cadres of study participants. The open-ended questions and probes had a primary focus of obtaining their experiences on reporting ADR based on their roles in the routine clinical practice as a doctor, pharmacist, or a nurse. The guides were prepared based on literature reviews [3, 12, 13], and the expertise of the investigators as the principal investigator (MK) is a pharmacist, while the coinvestigator (DLM) is a midwife, and the mentor (NS) is a public health specialist with a doctor of medicine background. The guides were initially developed in English and then translated into Kiswahili language. Before being used for data collection, the guides were translated back into English language to find discrepancy and confirm the accuracy of translation. We used Kiswahili guides to collect data because it is the most widely and comfortably spoken language for the participants and researchers. Before the start of data collection, the research team met to discuss and establish a common understanding of the data collection tools and the study's objectives. The data collection team comprised two researchers (1^st^ and 2^nd^ authors), a midwife, and a pharmacist with varying experience in conducting qualitative data collection and is involved in training nursing and pharmacy programmes. Both data collectors were fluent in Kiswahili and English. Researchers established rapport with each participant before the commencement of the interview. The interviews were conducted in a quiet room within a respective hospital chosen by the participant to ensure comfort and privacy. Each interview was conducted at a time convenient for the study participant based on a prearranged appointment with the researcher. All interviews were audio recorded in Kiswahili, and the notes were taken to complement the recorded information. Each interview lasted between 45 and 60 minutes.

### 2.5. Data Analysis

The thematic approach following Braun and Clarke guided the analysis of data [17]. Audio-recorded interviews were first transcribed verbatim. All authors first read the Kiswahili full transcripts and field notes to become familiar with the data and context. The analysis started by developing the initial codebook based on the study objectives, field notes, and interview guides. The codebook was refined from themes that emerged during the analysis. Data coding was performed by six researchers (EGP, AM, TM, NFK, BM, and CJS) who worked in pairs. To ensure reliability, the first two transcripts were coded by each group separately, and then they compared the codes for agreement on the final codes and coding. The generated codes were grouped into the respective predetermined codes through comparisons. Then, subthemes and themes were then generated from the general list of codes created. The focus was on broad patterns in the data, and the coded data were combined based on their relationships to form the themes. Lastly, themes were reviewed and discussed by all researchers, discrepancies were noted, and identified themes were finalized.

## 3. Results

Seven HCPs participated in the interviews, including three pharmacists, two doctors, and two nurses. Their ages ranged between 27 and 59. All three pharmacists hold a pharmacy bachelor's degree. One doctor had a master's degree in medicine, while the other held an advanced diploma in medicine. One of the two nurses holds a bachelor's degree, while the other holds a diploma in nursing. All of them had a work experience of more than 3 years.

**Figure 1:**
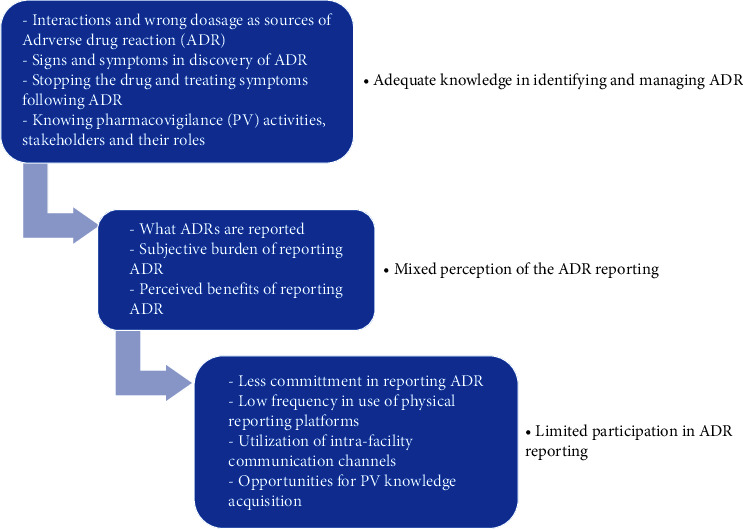
Summary of the findings.

Three themes were generated from the analysis of in-depth interviews (Figure 1), i.e., adequate knowledge in identifying and managing ADR, mixed perception in reporting ADR, and limited participation of HCPs in ADR reporting.

### 3.1. Theme 1: Adequate Knowledge in Identifying and Managing ADR

#### 3.1.1. Interactions and Wrong Medication Dosage as a Source of ADR

Participants stated that wrong medications and dosage given to patients by HCPs when treating a particular disease could result in ADR. The interviewee also emphasized that another source of ADR for some patients was the use of herbal remedies or expired medications. Participants also mentioned food-drug interactions and drug-drug interactions as causes for an individual to develop an ADR. Allergic reactions from specific drugs, such as penicillin antibiotics and medications containing sulfur were also mentioned.

*“For example, a patient may be given medication and suddenly start to experience hypersensitivity, and you find that he had asthmatic attacks, or you find out that the medicine gave him reactions… a patient with a history of allergy with sulfur may come to the facility but her allergic history was not taken and was given a medication containing sulfur and got reactions… there some foods that cause harm when taken along with certain medication, so we must treat a patient first and then look for the drug that caused the reactions” (Nurse, tertiary level facility)*

#### 3.1.2. Signs and Symptoms in the Discovery of ADR

Participants described the signs and symptoms associated with ADRs, such as difficulty in breathing, fever, elevated blood pressure, skin rashes, drowsiness, confusion, tearing, and peeling of the skin. Furthermore, the participants cited diarrhea associated with lopinavir use as the common ADR encountered by people living with HIV/AIDS when taking this medication. Also, the participants mentioned that ADR could worsen the patient's condition as it can cause convulsion and disability which is more fatal and life-threatening.

*“Yes, you may find that you have given medicine to someone else; the medicine you gave him may have started to cause a burning sensation, another may develop difficulty breathing, another may have rashes, another you may be surprised that you have given him medicine, and he may start to convulse immediately” (Nurse, secondary level facility)*

Also,

*“The very first way is to check the vital sign, that is, the vital sign is the one that will let you know that maybe the temperature has increased, or you find that the patient was fine when he suddenly got a fever, but when you gave him the fever, that means there are already reactions that have occurred, maybe you checked the blood pressure and it was fine. Maybe they can go down, maybe the heartbeat has gone up, so something unusual has entered it.” (Nurse, Tertiary level facility)*

#### 3.1.3. Stopping the Drug and Treating Symptoms following ADR

Participants elaborated on the steps involved in managing an ADR, including stopping the offending medication, switching to a different treatment regimen, and addressing any existing ADR symptoms to ensure patient safety. In addition, the participants stated that once ADR is discovered, effective communication with the prescriber and other HCPs should be done to ensure effective patient management, and ADR should be recorded to prevent its occurrence to the same patient in the future. Some participants said that any ADR encountered should be reported to the responsible authority via available ADR reporting platforms for the follow-up action.

*“It is often stopped immediately so that it does not continue to cause harm to the patient, but we will also send the report to the pharmacist so that they can determine whether the medication was administered after its expiration date or if there is a problem so that they can conduct additional monitoring” (Nurse, tertiary level facility)*

Also,

*“A person comes with an adverse drug reaction to the medicine, for example, at the beginning we were giving Efavirenz, they come and get a headache. Yes! We transferred many of them to Tenofovir* *+* *Lamivudi* *+* *Dolutegravir (TLD).” (PV focal person)*

#### 3.1.4. Knowing PV Activities, Stakeholders, and Their Roles

Participants were asked about the activities connected to PV. Their responses included counseling and educating patients on medication and their side effects, educating patients about ADR and ADR reporting, and providing regular reminders to HCPs about reporting ADRs. While other participants stated that important issues in PV are the availability of seminars on how to identify ADR, what to report, and how to report particularly on filling out ADR forms that are readily available at health facilities, some participants mentioned monitoring the quality of drugs and their storage conditions, reporting quality defects of poorly stored medicines, and training interns and new employees on ADR reporting as PV-related activities.

*“So, we teach them (HCPs) from the beginning how to report. If they fail, they come to the pharmacovigilance unit, and we help them report. We always pass onward to check if any ADR has occurred and the drugs themselves if they are of poor quality because they are part of pharmacovigilance. If they don't have a label if the expiration date has passed if they have not been properly maintained, we also report it” (PV Focal Person)*

The participants acknowledged that PV should not be an individual responsibility but rather a team effort involving all HCPs, the relevant regulatory body, and the council health management team members. Other participants described that the PV focal person should coordinate all PV-related activities, such as the assessment of ADR reporting and the provision of ADR-reporting tools. While other participants claimed that the coordination of PV activities in healthcare facilities must start with the pharmaceutical staff.

*“In my understanding, all those involved in the provision of healthcare services from nurses, and doctors to us pharmacists, must know and we are part of the people who are responsible in PV activities” (Pharmacist, secondary level health facility)*

Another participant perceived that nurses have more opportunity to identify and report ADRs and said the following:

*“What I can say is that ADRs reporting for a big percentage is a role of healthcare providers as the doctor prescribes the medicine, the pharmacist dispenses it and the nurse administers it. Since a nurse spends more time with the patient, he/she is able to observe the adverse reaction occurring to the patient since she/he spends more time with the patient. In this case, the nurse is responsible for reporting the ADR by completing the yellow form and submitting it to the appropriate authority” (Nurse, secondary level health facility)*

### 3.2. Theme 2: Mixed Perception of ADR Reporting

#### 3.2.1. What ADRs Are Reported

Participants perceived that the ADR to be reported includes any adverse events, unexpected ADRs other than those written on leaflets, or unwanted effects from medical devices. However, other participants acknowledged that all ADRs should be reported regardless of their severity, while others said that only severe ADRs and those interfering with major body systems should be reported.

*“As a pharmacist, I know that we have to report the side effects that the patient gets after using the drug. These are the ones we did not expect after taking the drug. There are those we expected …which we know are the side effects. Maybe after taking the drug the patient gets a stomach ache, but these are the ones that we did not expect to happen, so when it happens, we ask the patient to report it.” (Pharmacist, Secondary level facility)*

And

*“Basically, any reactions that the patient gets after using the drug and does not give relief to the patient, then that we should report.” (Pharmacist, Tertiary level facility)*

#### 3.2.2. Subjective Burden of Reporting ADR

Participants admitted that ADR reporting increases the workload and is not part of their job description, as it is tiresome and tedious work. Nevertheless, other participants reported that reporting ADR is simple, an integral part of their daily responsibilities, and does not increase their workload. They also emphasized that reporting is not an extra responsibility and does not require allowances.

*“That reporting is part of our responsibilities, which as HCP are supposed to do as part of my duties. I cannot say that it increases my workload, but it is something I am supposed to be ready to do… I don't believe in allowances because of dealing with many forms in clinical settings… this is our responsibility that we should work on because today I may see the reaction on the patient but tomorrow it will be a close person to me” (Doctor, tertiary level hospital)*

And

*“As I see it, it is a flow that as time goes by, things change first, in my opinion, I used to see it as one very heavy job and I used to see it as in our common language we say it is boring.” (Pharmacist, tertiary level hospital)*

#### 3.2.3. Perceived Benefits of Reporting ADRs

Participants highlighted the importance of reporting ADRs that facilitates the documentation of new ADRs and the withdrawal of medicine from the market. Participants expressed that ADR reporting helps in alerting the appropriate authorities for follow-up, interventions, and regulatory action. In addition, the participants explained how ADR reporting aids in risk group identification and prevention of ADR in vulnerable groups.

*“One of the benefits may be that there is a substandard drug in the market; therefore, if it (ADR) has occurred to a certain patient it is easier for TMDA (the relevant regulatory) to make follow-up if we report. After following up some drugs are banned. For example, during a certain period chloramphenicol injection was rejected … also during a certain period cloxacillin had several issues as per history and was removed from the market.” (Doctor, secondary level facility)*

Also,

*“Yes, it is important to report, because everyone has a problem, someone else can take care of the patient and gets different side effects, one happened here, another happened at a certain facility, so now, it appears that there is a problem, we will know that there will be some problem due to that particular drug that we are now reporting so that the relevant ministry can help us, if it can make alternative so that we can find another drug that will be able to help our customers and ourselves.” (Nurse, Tertiary level facility)*

### 3.3. Theme 3: Limited Participation in ADR Reporting

#### 3.3.1. Less Commitment in Reporting ADR

Some participants admitted that they had not completed ADR reporting forms or reported to the responsible authority. Also, some participants revealed that the reporting status of ADRs in their facilities varied from minimum to average as few ADRs were reported annually, while others acknowledged that they had not reported ADRs for more than two years. In addition, some participants mentioned that the HIV care and treatment clinic (CTC) accounted for the most reported ADRs.

*“The truth is the last time we sent that information (ADR reports) was in June 2020 if I am not mistaken … and it often this report came from CTC unit” (Pharmacist, secondary level facility)*

#### 3.3.2. Low Frequency in the Use of Physical Reporting Platforms

Participants reported that yellow forms are available and distributed at the health facilities for HCPs to report ADR, while green forms are also available for patients. However, the participants stated that not always they were committed to filling the yellow forms in case they observe ADR from patients.

*“Those forms are distributed CTC and, in all units, with inpatient care services because the training has been provided to HCP on ADR reporting therefore, we want the HCP in those departments to record any ADR that they come across for reporting purposes, though this is not done 100%” (Pharmacist, secondary level facility)*

Nevertheless, the participants reported to use available online platforms for reporting ADR. Also, the participants described that some online-reporting platforms are friendly to use and do not require the internet during the reporting process and no cost is involved. Furthermore, the participants described the use of normal phone text messages to report ADR to responsible authorities and the use of VigiFlow for uploading an ADR report.

*“You can report by using the electronic system that you can use the TMDA website straight away or by filling the forms or you can use the online systems in the mobile phone that number star one hundred and fifty star zero harsh then choose where to report therefore there are many different means” (Pharmacist, tertiary level health facility)*

#### 3.3.3. Utilization of Intrafacility Communication Channels

Participants acknowledged using various established channels for communicating ADR within healthcare facility, whereby some healthcare workers reported ADRs to the hospital pharmacist while patients reported to the nurse and the nurse reported to the doctor (prescriber). In addition, the participants said that ADRs are reported to the health facility management during clinical meetings.

*“First of all, if I am on the shift and my in charge (unit leader) when a patient gets the reactions, I must report to the in charge first because she/he is the overall in charge of what is happening in the ward…she (in charge) is also responsible for delivering the information to the doctor, so before I communicate with a doctor who prescribed the medicine I must inform the in charge of the ward.” (Nurse, tertiary level facility)*

#### 3.3.4. Opportunities for PV Knowledge Acquisition among HCPs

The participants outlined on-the-job training through scheduled hospital continuing medical education (CME) and seminars facilitated by the relevant regulatory authority as the major source of knowledge on PV and the related activities among HCPs. Other participants reported acquiring PV knowledge from medical college/university training and self-learning from various sources. In addition, other participants mentioned the media platforms as the source of information regarding PV.

*“The truth is that there is a little bit of self-study; sometimes, it is a form of training from time to time, and we always have on-the-job training among ourselves (HCPs). For those HCPs who know PV make presentations to other HCPs to make them understand, so we often have on-job training and self-study” (Pharmacist, tertiary healthcare facility)*

## 4. Discussion

We aimed to analyze the experiences of HCPs with PV and ADR reporting in the southern highlands of Tanzania. The study found that the HCPs had an adequate understanding of PV activities, the stakeholders involved, and their roles. Also, the participants expressed their experience with the sources of ADR, their identification, and the actions they took when encountering patients with ADR. We found mixed perceptions on what ADRs must be reported, whereby some participants were positive about reporting all ADRs because they knew the benefits of reporting, while others believed only serious ADRs should be reported. Nevertheless, the participants had mixed perceptions about the burden of ADR reporting, and despite knowing various platforms for reporting ADRs, we found there was limited participation of HCPs in ADR reporting.

Our findings revealed that the main actions taken by HCPs who were willing to report the ADR were stopping the suspect drug, treating ADR symptoms, and reporting to the authorities. The adequate knowledge about PV and its related activities observed among the participants in the current study could be due to the training and supportive supervision given by the national regulatory authority to HCPs to raise awareness and ADR reporting. Other studies reported that strengthening HCPs' knowledge was crucial to facilitating the reporting of ADRs in situations when they had limited access to information about PV and ADR reporting [18, 19]. Moreover, the fact that a focal person is available to oversee PV activities and provide supportive supervision may be the reason why HCPs are well informed about PV and ADR reporting. Supportive supervision and on-the-job training have been emphasized in the literature as essential to enhancing HCPs' clinical practice [3, 20].

Several platforms and tools for ADR reporting are available in Tanzania. Our study's findings show that both paper-based and electronic systems can be used for ADR reporting. According to the studies conducted in India and Ethiopia, the availability of reporting tools within health facilities improved ADR reporting. In these studies, the yellow and green forms were used for ADR reporting among HCPs and patients, respectively [21]. The participants in our study expressed more interest in the user-friendly electronic platforms than the paper-based ones, which were thought to be more complex and time consuming. This is consistent with findings from a study conducted in Ghana, which found that using electronic platforms enhanced timely ADR reporting [22]. To improve ADR reporting, user-friendly reporting methods must be available and known to stakeholders [23, 24].

Despite the availability of various ADR-reporting platforms, our study revealed that HCPs were less involved in ADR reporting. It was noted that although some of the HCPs acknowledged having reported very few ADRs to the appropriate authority, others had never reported any ADRs. The failure of the relevant authority to respond to previous ADR reports, a lack of motivation to report, and limited knowledge of what and how to report may have contributed to low ADR reporting. This is not unique to our findings; studies conducted in Northern Cyprus and Kenya also revealed that limited understanding of how to report an ADR and a lack of feedback prevented HCPs from reporting ADRs. As a result, these studies recommended providing feedback to encourage HCPs to report ADRs [25, 26]. Moreover, findings from Nepal and the United Arab Emirates (UAE) revealed that only 33.7% of the HCPs reported ADR during their clinical practices, while more than 60% did not know where and how to report ADR [27–29]. The underreporting of ADR may also be due to the limited engagement of pharmacists in clinical patient care. Therefore, to reduce ADRs and address the underreporting of ADRs, the focus should also be placed on clinical pharmacy services [30].

Similarly, the participants in our study acknowledged that reporting is crucial and beneficial for protecting the public's health [31, 32]. However, because of variations in knowledge and perception, some participants had differing thoughts about what type of ADRs are reported. The participants believed only serious ADRs needed to be reported, whereas others believed all ADRs needed to be reported. In Uganda, 91.9% of the HCPs felt that only serious and unusual ADRs must be reported [33]. This underscores the need for on-the-job training to enhance ADR-reporting skills [26].

Although our study and other studies findings indicate that all PV stakeholders, including HCPs, regulatory authorities, and consumers or patients, were accountable for ADR reporting [21, 34]. In line with findings from other studies [18, 24, 29], our findings demonstrate that reporting ADRs was perceived as a tiresome, time-consuming, and subjective burden that required a monetary incentive. However, some HCPs believed that ADR reporting was part of their duties that did not require allowances, similar to what was reported by Sharrad et al. [35]. Evidence points out the use of various motives by HCPs as a strategy to increase ADR reporting, such as monetary incentives, appreciation letters, and congratulatory notes posted on notice boards [36].

To tackle the knowledge gap and improve ADR reporting, our findings have shown the need to strengthen both preservice and in-service training. We discovered that some HCPs did not receive training during their college or university education program, which emphasizes the importance of including PV in the medical curriculum. The improvement in ADR reporting in the Netherlands resulted from including the PV content in undergraduate medical training programs [37]. Due to differing coverage during their medical training, other studies found that pharmacists had a greater understanding of ADRs than doctors and nurses [25]. Therefore, there is a need to continue providing regular training to ensure that all HCPs have the necessary skills to use the ADR-reporting platforms [31].

### 4.1. Similarities and Differences Observed across Participants

The current study interviewed participants from different medical professions, including doctors, pharmacists, and nurses. Following the analysis, we observed that both participants displayed adequate knowledge of PV and ADR, but pharmacists were more aware of ADR-reporting platforms. Doctors and nurses perceived that ADR reporting should be championed by pharmacists, who must be engaged in patients' primary care to increase ADR reporting. The medical curriculum could influence the discrepancy observed as pharmacists are taught to deal with medications and medical device-related issues, while medical doctors' and nurses' curricula focus on diagnosis, treatment, and taking care of patients. In addition, pharmacists are aware of the ADR reporting because the PV and drug information unit are under the Department of Pharmacy and the PV focal person is also a pharmacist. Nevertheless, participation in ADR reporting is poor among the participants.

### 4.2. Methodological Considerations

In our article, we discuss methodological considerations in two parts as follows: the first part discusses study limitations and mitigations, while the second part discusses trustworthiness. Social desirability may provide some limitations to our findings since data collection was led by two researchers who are involved in training nursing and pharmacy programmes, and HCPs may have felt compelled to provide desired responses rather than truthful ones. However, the fact that data collectors have adequate probing skills and are not very senior as compared to the participants offsets this limitation. We discuss trustworthiness through Guba's four criteria of credibility, dependability, conformability, and transferability [38]. The credibility of this study's findings was enhanced through member checks and the triangulation of study participants (doctors, nurses, and pharmacists). The triangulation of study settings (two healthcare facilities) and researchers enhanced the credibility and dependability of this study. To confirm that the findings reflected the perspectives of the participants and not the researchers' understanding of the research topic, themes were generated inductively using thematic analysis and presented with the support of subthemes and succinct quotes. The transferability of this study's findings is enhanced by describing the study setting, context, population, data collection methods, guides, process, and analysis [39].

## 5. Conclusion

The findings of this study describe the experiences of HCPs in reporting ADRs. Although the participants had adequate understanding of PV and ADR reporting, fewer HCPs participated in ADR reporting because they perceived the process as challenging, time consuming, and subjectively burdensome. These reported experiences suggest that reporting platforms, supportive supervision, and training must be improved to increase ADR reporting. In collaboration with health facilities, the national regulatory authority should explore and identify the friendliest reporting alternatives that can motivate HCPs to report ADRs. Furthermore, we recommend further study to establish the health training institutions' readiness to harmonize the training curricula and include the content of PV and ADR reporting.

## Data Availability

The datasets used to support the findings of this study are available from the corresponding author upon reasonable request

## Additional Points

*Key Points*. (i) Healthcare providers demonstrated adequate knowledge on pharmacovigilance and adverse drug reactions. (ii) Healthcare providers demonstrated good perception on benefits of adverse drug reaction reporting, but some perceive reporting as an extra work which requires allowances. (iii) Healthcare providers reported low participation in adverse drug reaction-reporting activities. *Plain Language Summary*. Pharmacovigilance (PV) is the process of keeping an eye on how safe medicines and medical devices are. This takes commitment from all the parties involved. Healthcare providers (HCPs) are one of the most important people who have a role in pharmacovigilance and reporting adverse drug reactions (ADRs). The literature says that a HCP's work is based on what they know and how they see things. In Tanzania, the people in charge have done a number of things to increase the number of reports. Among the things that have been done are making rules and regulations, putting out an electronic reporting system, providing on-the-job training to the HCPs, and appointing a PV focal person in each zone. Even with all the attempts, only a small number of ADRs are reported. Therefore, we conducted a qualitative study to explore the experiences of HCPs in PV and ADR reporting. The study found that HCPs know about PV activities, ADRs, what to do when they come across an ADR, and how to report an ADR. The participants also had a good impression of the benefits of reporting ADRs, but some saw it as an extra work that needed to be rewarded. Lastly, the study found that HCPs do not perform well in reporting ADRs. We suggest that the authority set up incentives and make sure that everyone has access to the framework for reporting ADRs.
